# Redox gradients define the ecological niche of ciliates with denitrifying endosymbionts in anoxic lake waters

**DOI:** 10.1093/ismejo/wrag043

**Published:** 2026-03-01

**Authors:** Linus M Zeller, Sina Schorn, Louison Nicolas-Asselineau, Jakob Zopfi, Soeren Ahmerkamp, Carsten J Schubert, Fabio Lepori, Marcel M M Kuypers, Jon S Graf, Jana Milucka

**Affiliations:** Department of Biogeochemistry, Max Planck Institute for Marine Microbiology, 28359 Bremen, Germany; Department of Biogeochemistry, Max Planck Institute for Marine Microbiology, 28359 Bremen, Germany; Department of Marine Sciences, University of Gothenburg, 405 30 Gothenburg, Sweden; Department of Biogeochemistry, Max Planck Institute for Marine Microbiology, 28359 Bremen, Germany; Department of Environmental Sciences, University of Basel, 4056 Basel, Switzerland; Department of Biogeochemistry, Max Planck Institute for Marine Microbiology, 28359 Bremen, Germany; Leibniz Institute for Baltic Sea Research, 18119 Rostock, Germany; Department of Surface Waters-Research and Management, Swiss Federal Institute of Aquatic Science and Technology (EAWAG), 6047 Kastanienbaum, Switzerland; Institute of Biogeochemistry and Pollutant Dynamics, ETH Zurich, 8092 Zurich, Switzerland; Institute of Earth Sciences, University of Applied Sciences and Arts of Southern Switzerland (SUPSI), 6850 Mendrisio, Switzerland; Department of Biogeochemistry, Max Planck Institute for Marine Microbiology, 28359 Bremen, Germany; Department of Biogeochemistry, Max Planck Institute for Marine Microbiology, 28359 Bremen, Germany; Department of Biogeochemistry, Max Planck Institute for Marine Microbiology, 28359 Bremen, Germany

**Keywords:** protists, meromictic lakes, endosymbiosis, anoxic, ciliates

## Abstract

Bacterial endosymbionts of the family *Candidatus* Azoamicaceae obligately associate with anaerobic ciliates belonging to the class Plagiopylea. The symbionts’ unique role for their host involves anaerobic respiration of nitrate and generation of adenosine triphosphate (ATP), analogous to the role of mitochondria in aerobic eukaryotes. As this symbiosis remains so far uncultured, insights into its functioning have been mainly inferred from environmental metagenomes. Here, we investigated the distribution and environmental role of this symbiosis in the anoxic basins of two freshwater lakes, Zug and Lugano (Switzerland), over a course of several years. We found that the environmental niche of the ciliate host is defined by the combined effects of sulfide, oxygen, and nitrate, the latter of which is essential for the symbiont’s respiratory function. Moreover, the distribution and abundance of ciliates with denitrifying endosymbionts in the water column suggest that they may substantially contribute to nitrate consumption in Lake Zug. Our microscopic analyses further demonstrated a coordinated division of the *Ca.* Azoamicus ciliaticola symbionts and their ciliate hosts, implying a vertical inheritance of denitrifying symbionts. These observations offer new insights into the evolution of ciliates with denitrifying endosymbionts and their ecological role in oxygen-depleted lake waters.

## Introduction

Ciliates comprise a diverse and versatile group of unicellular eukaryotes belonging to the phylum Ciliophora. As predators of bacteria and other protists, they play a key role in the proper functioning of aquatic ecosystems and food webs [1]. Their ability to establish symbiotic relationships with prokaryotic partners is thought to be crucial for their survival in a wide variety of habitats, particularly in those with low or no oxygen (e.g. [2]).

Ciliates move temporarily or permanently into oxygen-depleted waters, either due to physiological sensitivity to oxygen [3] or to exploit food resources that are inaccessible to aerobic competitors [2]. In the absence of oxygen, anaerobic ciliates typically perform fermentation and often associate with methanogenic archaea, which consume the hydrogen produced during fermentation and thereby increase the energy yield of the reaction [2]. Ciliates of the genus *Loxodes* have been reported to perform denitrification under anoxic conditions, utilizing a nitrate reductase located in the mitochondrial membrane, while retaining the ability to respire oxygen when it becomes available [4]. Other ciliates, belonging to the obligately anaerobic class Plagiopylea, have evolved yet another mechanism to survive in anoxic environments. They acquired obligate bacterial endosymbionts, which perform nitrate respiration and provide the host with energy in the form of adenosine triphosphate (ATP) [5]. The endosymbionts, belonging to two genera *Candidatus* Azoamicus and *Candidatus* Azosocius, within the *Ca*. Azoamicaceae family have highly reduced genomes (246–374 kbp) that have retained genes essential for energy production through denitrification and the exchange of ATP with its host [5–7]. The dedicated role of these bacterial symbionts for host respiration makes this symbiotic relationship a functional analogue to the mitochondria [5]. These organelles are integral for the physiology of all aerobic eukaryotes, and they fulfill a diverse range of functions in eukaryotic cells [8]. In anaerobic protists, mitochondria can persist as so-called mitochondria-related organelles (MROs), in a spectrum of states of genome and structural reduction, with the most commonly documented forms being hydrogenosomes [9, 10]. However, unlike these “true” organelles, *Ca*. Azoamicaceae symbionts still retain a greater number of genes than mitochondria, suggesting a later acquisition and shorter co-evolution with their host. The degree of these symbionts’ integration into the host cells, in terms of gene and protein transfer, remains unclear. However, they seem to represent a stage on the evolutionary path between obligately vertically-inherited endosymbionts and “true” cell organelles.

Plagiopylean ciliates hosting *Ca*. Azomicaceae symbionts were first discovered in the anoxic bottom waters of the permanently stratified Lake Zug in Switzerland [5]. Even though freshwaters were assumed to represent a dominant habitat of the *Ca*. Azoamicaceae symbionts [6], with sequences retrieved from other meromictic lakes, e.g. Lake Pavin (France) [5], the original study remains to date the only report of a respiratory symbiosis between ciliates and bacterial symbionts from a lacustrine environment. Closely related endosymbionts have been identified in diverse other environments, such as groundwater and wastewater, where they are hypothesized to serve a similar respiratory role for their hosts [6, 7]. Many of the *Ca*. Azoamicaceae symbionts encode the genetic potential for aerobic respiration in the form of a cytochrome-*cbb_3_* oxidase [6, 7]. However, *Ca*. Azoamicus ciliaticola from Lake Zug lost these genes and is only capable of an obligately anaerobic lifestyle [5]. The demonstrated ability of these ciliate-associated symbionts to reduce nitrate to dinitrogen gas makes them potential contributors to nitrogen loss in anoxic waters, a role that is typically attributed to free-living prokaryotes [5].

Respiratory *Ca*. Azoamicaceae symbionts and their hosts so far remain uncultured. Thus, to better understand the factors governing their distribution and environmental role, we investigated environmental populations of these ciliates in Lake Zug across 6 years. Additionally, we describe the occurrence of this symbiosis in the south alpine Lake Lugano (Switzerland), which contains euxinic (i.e. anoxic and sulfidic) bottom waters. Our data reveal how the environmental niche of the lacustrine plagiopylean ciliates harboring denitrifying endosymbionts is determined by redox conditions. Finally, the occurrence of dividing hosts in both lakes allowed us to demonstrate vertical inheritance of these denitrifying symbionts.

## Material and methods

### Water column profiling and sample collection

Between October 2018 and July 2023, we conducted 10 sampling campaigns in Lake Zug (Switzerland) encompassing all seasons. Our sampling site was situated in the deep meromictic southern basin, which had previously been identified as a habitat for ciliates hosting *Ca.* A. ciliaticola endosymbionts [5]. At this site, the water column is ~200 m deep. Oxygen and temperature depth profiles were recorded with a CTD60 (Sea&Sun Technology) from October 2018 to July 2021, equipped with a Clark-type oxygen sensor (accuracy ±3%, resolution 0.1%) to record oxygen profiles as conducted previously [5]. In the following campaigns, profiles were recorded using an RBRconcerto [3] CTD (RBR Ltd) with an integrated dissolved oxygen sensor (accuracy ±2%, resolution 1%).

In August 2020, a single sampling campaign to Lake Lugano was conducted. Water column samples were collected from the 288 m-deep northern basin, where the water exhibits a stable anoxic hypolimnion below 100–130 m [11, 12]. Oxygen and temperature were recorded with a CTD (Sea&Sun Technology) with an integrated dissolved oxygen sensor (accuracy ±2%, resolution 1%).

From both lakes, discrete water samples were collected for chemical and molecular analyses with 5 l Niskin bottles in regular intervals of 5–10 m, starting from ca. 10 m above the oxic–anoxic interface to ca. 190 m water depth in Lake Zug and 250 m in Lake Lugano. These sampling depths were chosen to achieve high-resolution profiles within the anoxic water column.

As the sensors are not sensitive enough to detect trace-amounts of oxygen, the oxic–anoxic interface was defined as the depth below which oxygen concentrations remained at a constant lowest value. Oxygen measurements were conducted in intervals of 250–3000 ms.

### Nitrate and nitrite measurements

Samples for nitrate and nitrite concentration measurements were taken from all depths sampled during each campaign in Lake Zug and one sampling campaign in Lake Lugano (August 2020). For nitrate and nitrite measurements, 30 ml of lake water was sterile-filtered through a hydrophilic, low-binding polyethersulfone 0.22 μm pore size syringe filter with a low amount of extractables (Millex Millipore) and frozen at −20°C. Concentration measurements were performed with a commercial QuAAtro Segmented Flow Analyzer (SEAL Analytical).

### Sulfide measurements

Sulfide concentrations were determined for all water depth samples in Lake Lugano in August 2020. For sulfide measurements, 10 ml unfiltered lake water was fixed with 1 ml of a 5% w/v zinc chloride solution. Sulfide concentration measurements were performed using the methylene blue method described previously [13].

### Fluorescence *in situ* hybridization

Plagiopylid ciliates harboring *Ca*. Azoamicaceae endosymbionts were identified by fluorescence *in situ* hybridization (FISH) using an oligonucleotide probe [eub62A3_813 (5′-CTAACAGCAAGTTTTCATCGTTTA-3′)], terminally double-labelled with Atto488 dye, specific to *Ca*. A. ciliaticola as previously described [5]. For that, 500 ml lake water was fixed with 1.5% final concentration paraformaldehyde and kept cold until filtration onto polycarbonate filters (3 μm pore size, Merck Millipore), latest within 4 h after sampling. Filters were stored at −20°C. The abundance of plagiopylids was determined in all samples taken from Lake Zug and in all samples taken from Lake Lugano in August 2020.

To identify the ciliate host in Lake Lugano, ciliates were additionally hybridized with a specific plagiopylean 18S ribosomal ribonucleic acid (rRNA) probe [plagi_1083 (5’-TTGTGTCCATACTTCCCCC-3′)], terminally double-labelled with Atto594 dye, as previously described [7] (see also Supplementary Methods). Ciliates were counterstained with 4′,6-diamidino-2-phenylindole (DAPI). Ciliates were counted on filter sections sized at 1/16 of the original filter (32 mm effective filter diameter) using an Axio Imager 2 microscope (Zeiss, 40× objective). All labeled ciliates on the filter section were counted and ciliate numbers were then extrapolated to estimate the total number across the entire filter area and corresponding filtered water volume. Ciliates were regarded as dividing if their cell bodies and macronuclei were visibly elongated or already separated based on the DAPI staining. For imaging, image stacks were recorded on a confocal laser scanning microscope (Zeiss LSM 780, 63× oil objective, 1.4 numerical aperture).

### Abundance of prokaryotes and small protists

In May 2022, additional samples were collected from Lake Zug, to determine the abundance of free-living prokaryotes and small protists. For prokaryote analysis, 10 ml of lake water was collected from each depth. For small protist analysis, 50 ml of lake water was collected. Samples were fixed with 2% paraformaldehyde and kept cold until filtration onto polycarbonate filters (0.22 μm pore size, 17 mm effective filter diameter; Merck Millipore) latest within 4 h after sampling. Filters were stored at −20°C. Prokaryotes were counted using an Axio Imager 2 microscope (Zeiss, 100× objective) on filter sections that were embedded in 0.2% MetaPhor Agarose and counterstained with DAPI for 10 min at 4°C in the dark. The samples were then mounted in antifade medium composed of Vectashield and Citifluor at a ratio of 4:1 (Vector Laboratories). Prokaryotic cell counts were conducted using a counting grid (125 × 125 μm), counting at least 10 fields of view with a minimum of 1000 cells per filter.

The abundance of small protists (i.e. smaller than 20 μm) was determined on filter sections hybridized with eukaryote-specific horseradish peroxidase–labelled FISH probes (EUK 1209 (5’-GGGCATCACAGACCTG-3′), EUK 502 (5’-ACCAGACTTGCCCTCC-3′), EUK 309 (5’-TCAGGCTCCCTCTCCGG-3′), EUK B (5’-TGATCCTTCTGCAGGTTCACCTAC-3′) [14] with the unlabeled competitor probe KIN 516 (5′ -ACCAGACTTGTCCTCC-3′) [15]) at a formamide concentration of 40% (see Supplementary Methods). Prior to use, all probes were tested using TestProbe version 3.0, with the SSU r138.2 SILVA database with the REFNR sequence collection (https://www.arb-silva.de/testprobe, released 11 July 2024) to assess how much of the eukaryotic diversity the probe mix covers. With zero weighted mismatches, excluding sequences containing N bases, the probes EUK_1209 and EUK_502 cover each over 88% of the diversity of 18S rRNA gene sequences in the database (89.9%, respectively, 88.2% coverage, see also Supplementary Files 2–5). At least 20 fields of view were counted per filter. To exclude nonspecific binding or insufficient inactivation of endogenous peroxidases, the probe Non388 (5’-ACTCCTACGGGAGGCAGC-3′) was used on separate filter pieces.

### Correlation analysis

A linear correlation analysis was conducted between plagiopylid abundance and the abundance of small protists and free-living bacteria, respectively. A further linear correlation analysis was conducted between plagiopylean 18S rRNA operational taxonomic units (OTUs) relative abundance and *Ca*. Azoamicus 16S rRNA OTU relative abundance. The Pearson coefficient was calculated and tested for significance using a two-sided permutation test with 20 000 iterations with the perm.cor.test function of the package jmuOutlier v2.2 [16] in R studio [17] (Table S1, Supplementary File 1). This nonparametric test was used, as data were not normally distributed. Significance was tested against an α-value of 0.05.

### Nucleic acid extraction, metagenome sequencing, and genome assembly of Lake Lugano water samples

In Lake Lugano, 4 l of lake water was sampled for DNA analyses from three selected water depths (90, 100, and 130 m), on 19^th^ of August 2020. The lake water was filtered onto 0.22 μm filter cartridges (Sterivex, Merck Millipore), using a peristaltic pump directly on site. Filters were immediately frozen and stored at −80°C until nucleic acid extraction. Nucleic acid extraction was performed using the DNeasy PowerWater Kit (Qiagen) according to the manufacturer’s instructions, including the alternative lysis step (10 min at 65°C after adding solution PW1). Sequencing with a HiSeq 2500 System (Illumina) was conducted as described in [5]. The metagenomic reads were trimmed with Trimmomatic v. 0.39 [18] with the parameters “ILLUMINACLIP:TruSeq3-PE.fa:2:30:10 LEADING:3 TRAILING:3 SLIDINGWINDOW:4:10 MINLEN:50”. Reads were assembled using metaSPAdes v. 3.14.1 [19] and k-mer lengths of 21, 33, 55, 77, 99, and 127. The contigs were subsequently binned with MetaBAT2 [20] using the following options: minContig 2000, minCV 1.0, minCVSum 1.0, maxP 95%, minS 60, maxEdges 200, and minClsSize 200 000. A taxonomic classification was assigned to each bin using GTDB-Tk v. 1.0.2 [21] and the classify_wf command. One bin consisting of a 292 625 bp-long contig was identified as *Ca*. Azoamicus. The contig ends were blasted against each other in the online BLAST tool [22, 23], and the overlapping fragments were removed, resulting in a single 292 498 bp-long contig. The circular contig was reoriented by blasting it against the *Ca*. A. ciliaticola genome (PRJEB27314). Average nucleotide identities (ANIs) of the metagenome-assembled genome (MAG) from Lake Zug (GCF_902860225.1) [5] and Lake Lugano (retrieved in this study) were calculated using the webservice JSpeciesWS [24].

### Primer design, polymerase chain reaction, and Sanger sequencing

Specific primers for the ciliate class Plagiopylea were designed using the probe design tool in ARB version 6.1 [25] and the SILVA SSU Ref NR 99132 database (https://www.arb-silva.de/download/arb-files/) including 48 sequences of plagiopylean 18S rRNA genes. The primer pair plagi_289_F (5′-TCAAGTTTCTGCCCTATCAC-3′) and plagi_1107_R (5′-TCAGACTTGTGTCCATACTT-3′) binds specifically only to members of the class Plagiopylea, with weak mismatches to some Trimyemidae sequences (for a detailed description of the primer pairs, see Supplementary Results).

The specific annealing temperature of the primer pair was experimentally tested in a gradient polymerase chain reaction (PCR) as described in the Supplementary Methods. At temperatures above 58°C, the intensity of the PCR band visibly decreased (Fig. S1). For all the following PCRs, the primer pair was used at an annealing temperature of 58°C.

To amplify the 18S rRNA gene sequence of plagiopylean ciliates from Lake Lugano, the primer pair plagi_289_F/plagi_1107_R was used in a PCR on DNA extracts of water samples from 90, 100, and 130 m depth. The PCR scheme followed the one described for the gradient PCR (see Supplementary Methods). The PCR was conducted with the premixed PCR Mastermix 2× (Thermo Fisher Scientific) for 35 cycles. PCR products of ca. 800 bp length were amplified from 100 and 130 m depth. There was no band visible in DNA extracted from 90 m depth.

The PCR products from 100 and 130 m depth were prepared for Sanger sequencing as described previously [26] (see also Supplementary Methods). The resulting partial sequences were aligned with the 18S rRNA gene of the Plagiopylean host of *Ca*. A. ciliaticola Zug (Genbank: LR798089.1 [5]) using blastn [22, 23].

### Amplicon analysis of Lake Lugano

Amplicon sequencing data from nine campaigns from 2015 to 2019 from Lake Lugano were retrieved previously [12] and accessed from the National Center for Biotechnology Information (NCBI) sequence read archive (for accession numbers and further information, see Table S4). PCR amplification conditions, library generation, and analysis of 16S rRNA gene amplicons are described in detail in [12]. 18S rRNA gene amplicons were analyzed as described in the Supplementary Methods.

### Small subunit rRNA gene phylogeny

The sequences used for 16S (prokaryotic) and 18S rRNA (eukaryotic) gene phylogeny are described in the Supplementary Methods. Sequences were aligned with local pair alignment (1000 cycles of iterative refinements) using MAFFT version 7.525 [27]. A maximum likelihood phylogenetic tree was calculated using IQTREE version 2.3 [28]. The models for sequence evolution were selected using ModelFinder (best-fit model 16S rRNA gene phylogeny: TVM + F + I + R2; best-fit model 18S rRNA phylogeny: GTR + F + I + R3) [29], bootstraps (1000 replicates) were calculated using UFBOOT2 [30]. The tree was visualized in iTOL version 6.9 [31].

### Contribution of plagiopylids to nitrogen loss in anoxic lake waters

The data and calculations are shown in Table S5.

Total volumetric plagiopylid denitrification rate for depth *i* (*R*_*p,i*_):


$${R}_{P,i}={N}_{P,i}\ast{R}_{SP}$$

where *N_P_* is the number of plagiopylids in depth *i*, and *R_SP_* is the denitrification rate per plagiopylid as determined previously [5] (12 pmol N d^−1^ ciliate^−1^).

Total volumetric plagiopylid-specific denitrification flux for depth *i* (*J_P,i_*):


$${J}_{P,i}=\frac{\left({R}_{P,i}+{R}_{P,i+1}\right)}{2}\ast \Delta{H}_j$$

where Δ*H_j_* is the depth difference between depth *i*, and *i* + 1.

Nitrate flux for depth *i* (*J_NO3,i_*):


$${J}_{N{O}_3,i}=D\ast \Delta{C}_{N{O}_3,i}$$

where *D* is the diffusion coefficient taken from [32] (27*10^−6^ m^2^ s^−1^), and Δ*C_NO3,i_* is the nitrate concentration difference between depth *i*, and *i* + 1.

Plagiopylid contribution to nitrogen loss for depth *i* (*R_P,i_*):


$${R}_{P,i}=\left(\frac{J_{P,i}}{J_{N{O}_3,i}}\right)\ast 100\%$$

### Sulfide sensitivity assays

For sulfide sensitivity assays, anoxic water samples were collected in July 2023 from the depth showing the highest ciliate abundance (as determined by FISH) using 5 l or 20 l Niskin bottles. The water was transferred into 2 l glass bottles using gas-tight tubing, filling the bottles slowly from the bottom to top. The bottles were allowed to overflow with lake water to minimize oxygen exposure of the water during filling. Bottles were closed headspace-free with deoxygenated butyl rubber stoppers and stored at 4°C in the dark until experiments were performed, within a month after sampling.

We investigated the sulfide sensitivity of ciliates using a capillary-based experimental setup similar to that previously described for aerotaxis assays [5]. All experiments were conducted in a temperature-controlled room at 4°C and within a glove bag (AtmosBag, Aldrich) flushed with nitrogen gas to maintain anoxic conditions. Ciliates were individually isolated after enrichment through gravity flow on a 5 μm polycarbonate filter (Merck Millipore); then, ~15 to 20 ciliates were collected in a droplet of sterile-filtered anoxic lake water. These were transferred into a glass capillary tube with a 1.3 mm inner diameter by capillary action and a 1% agarose (Biozym LE Agarose) plug saturated with sulfide solution at different concentrations (10 and 100 μM sulfide) was placed at one end of the capillary, while the other end was sealed with parafilm. Then, the capillary was placed inside an Exetainer (Labco) prefilled with helium-purged lake water. The exetainer containing the capillary was then mounted onto a custom-built long-distance microscope, and ciliates were observed for 5–15 min.

## Results

### Inter-annual distribution of anaerobic plagiopylids harboring *Ca*. Azoamicus ciliaticola in the anoxic hypolimnion of Lake Zug

**Figure 1:**
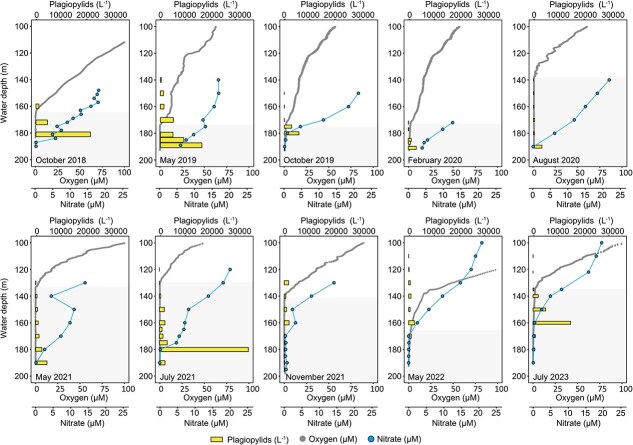
Water column distribution of *Ca.* Azoamicus ciliaticola–harboring plagiopylids, as well as oxygen (dotted line) and nitrate (dots) concentrations in the anoxic hypolimnion of Lake Zug. Profiles were recorded during 10 sampling campaigns between 2018 and 2023. Plagiopylid counts (bars) were determined by fluorescence *in situ* hybridization using probe eub62A3_813. Shaded areas indicate anoxic water depths.

We investigated the inter-annual distribution of anaerobic ciliates of the class Plagiopylea (hereafter: plagiopylids) harboring *Ca.* Azoamicus ciliaticola endosymbionts in the bottom waters of Lake Zug during 10 sampling campaigns between 2018 and 2023 (Fig. 1). During this period, Lake Zug exhibited a persistently anoxic hypolimnion, with the depth of the oxic–anoxic interface varying from 187 m in October 2019 to 131 m in July 2023, thus constraining the anoxic bottom waters to a variable thickness of ca. 10–70 m. During the 10 sampling campaigns, nitrate was present in the anoxic waters, although the maximum depth of detection varied between years.

Plagiopylid hosts of *Ca*. A. ciliaticola were identified by FISH using oligonucleotide probe eub62A3_813 targeting the 16S rRNA gene of *Ca*. A. ciliaticola. Plagiopylids with distinctive endosymbiont-specific fluorescent signals were observed in every campaign, nearly exclusively in the water samples from the anoxic hypolimnion. These ciliates were of uniform spheroid shape and size (average volume of 3658 μm^3^; *n* = 60) and exhibited green autofluorescence. Thus, they could be clearly differentiated from other protists in the water column (see also Fig. S2 and Supplementary Results).

Between October 2018 and July 2021, the abundance of plagiopylids peaked at or below 180 m depth (ranging from 3136 plagiopylids l^−1^ in February 2020 to 34 944 plagiopylids l^−1^ in July 2021), thus well below the oxic–anoxic interface where oxygen was not detected during any sampling campaign. In fact, plagiopylids were typically absent from oxic waters with oxygen concentrations above 10 μM. The highest abundance of plagiopylids was consistently observed at depths where oxygen was absent and nitrate concentrations were low but still detectable (between 0.1 and 5.7 μM), whereas plagiopylids were mostly absent from deeper, nitrate-depleted waters. From November 2021 onwards, the vertical distribution of plagiopylids shifted, with the highest abundances at depths around 160 m (ranging from 1888 plagiopylids l^−1^ in November 2021 to 14 816 plagiopylids l^−1^ in July 2023). This coincided with shallower nitrate gradients and concentrations decreasing below 1 μM already at 160 m.

To investigate additional factors that could influence plagiopylid distribution, such as the availability of food sources, we collected further samples from Lake Zug in May 2022 to assess the abundance of prokaryotes and small protists. Like the plagiopylids, prokaryote abundance increased with depth between 120 m (100 μM oxygen) and 160 m (4 μM oxygen). Smaller protists were less abundant at depths with high plagiopylid numbers (Fig. S3). There was however no significant linear correlation between the abundance of these potential food sources and plagiopylid abundance (Table S1).

### Presence and diversity of *Ca*. Azoamicus symbionts in Lake Lugano

**Figure 2:**
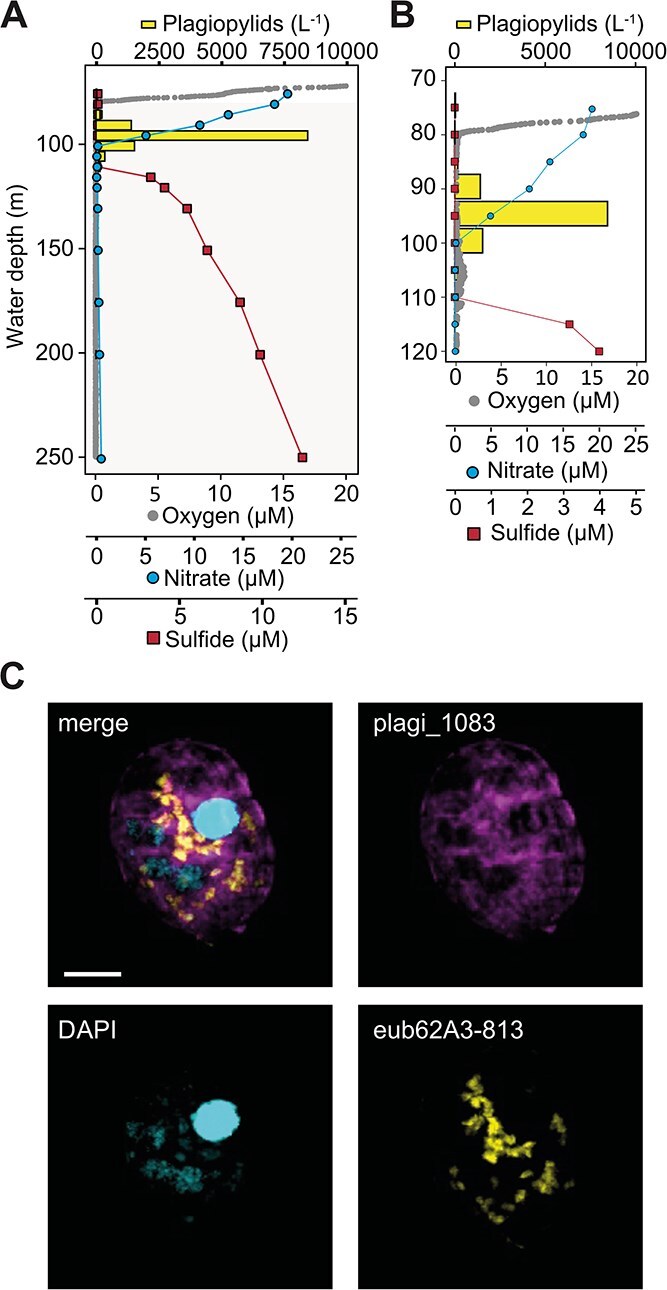
Distribution and visualization of *Ca.* Azoamicus ciliaticola–harboring plagiopylids in Lake Lugano. (A) Water column profiles of oxygen (dotted line), nitrate (dots), and sulfide (squares), and plagiopylid cell counts (bars) recorded in August 2020 in the 288 m deep northern basin. Plagiopylid counts were determined by fluorescence *in situ* hybridization using probe eub62A3_813. Shaded areas indicate anoxic water depths. (B) Plagiopylids with endosymbiont-specific fluorescent signals are present in a narrow zone between the oxic–anoxic interface and deeper, sulfidic waters. (C) FISH images from 95 m water depth from the northern basin of Lake Lugano showing simultaneous fluorescence labeling of plagiopylean ciliates using the 18S rRNA probe plagi_1083 and *Ca*. Azoamicus using the 16S rRNA probe eub62A3_813. Ciliate nuclei were counterstained with DAPI. Image stacks were recorded with a confocal laser scanning microscope and are presented as maximum intensity projection. Scale bar, 10 μm.

Available molecular data indicated the presence of *Ca*. Azoamicus–related organisms in other stratified freshwater lakes [5]. To study these organisms in another lake, with a different hydrochemistry (i.e. sulfidic bottom waters), we sampled Lake Lugano, a 288 m-deep lake located at the border between Switzerland and Italy. The lake is in geographic proximity to Lake Zug, but the two lakes are separated by the Alps, making an exchange of water between them very unlikely. In August 2020, water samples were collected from the northern basin of Lake Lugano between 75 and 250 m depth. At the time of sampling, oxygen was depleted below a water depth of ~80 m and nitrate was detected down to a depth of 95 m (Fig. 2A). Sulfide was detected in waters below 110 m, increasing from 3 μM at 115 m to 12 μM at 250 m depth. These profiles resulted in a narrow anoxic, nitrate-replete, sulfide-free water layer between 85 and 110 m depth (Fig. 2B).

**Figure 3:**
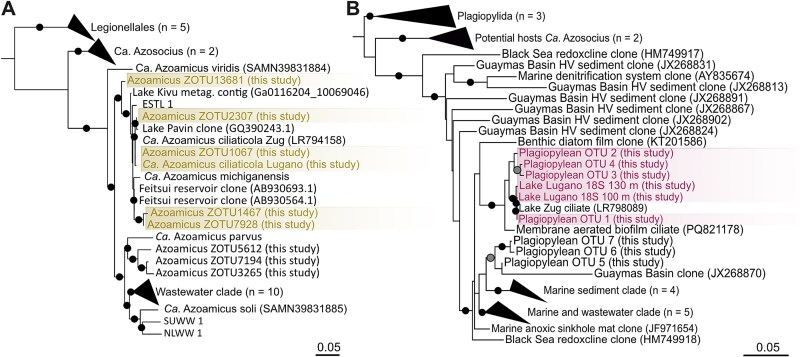
Phylogeny of respiratory endosymbionts and their hosts in Lake Lugano. (A) 16S rRNA gene sequence–based maximum likelihood phylogenetic tree of *Ca*. Azoamicus from Lake Lugano metagenomes and amplicon sequences of related gammaproteobacterial orders and environmental clades. The closest relatives to *Ca*. A. ciliaticola Zug from this study are highlighted. (B) Ciliate 18S rRNA gene sequence–based maximum likelihood phylogenetic tree of the class Plagiopylea. Sequences of ciliates from Lake Lugano closest related to the plagiopylean ciliate from Lake Zug are highlighted. Bootstrap values for both trees are shown as circles at the respective nodes and indicate bootstrap support of >80% (grey) and >90% (black) out of 1000 resamplings. Scale bars indicate 0.05 nucleotide substitutions per site. The full trees are shown in Supplementary Figs S5 and S6. Additionally, a 16S rRNA phylogeny, with a collapsed clade of the host of *Ca*. A. ciliaticola is shown in Supplementary Fig. S7.

We conducted metagenome analysis on DNA retrieved from 90, 100, and 130 m depth in Lake Lugano in August 2020. We obtained a metagenome-assembled genome (MAG) (see Material and Methods) from 100 m depth, containing a full 16S rRNA gene sequence 99.7% identical to *Ca*. A. ciliaticola from Lake Zug (GCF_902860225.1) [5]. Closely related 16S rRNA gene sequences were recovered from two other anoxic lakes, Lake Kivu (Democratic Republic of the Congo/Rwanda; accession number: Ga0116204_10069046) and Lake Pavin (France; accession number: GQ390243.1) [33], as well as from the Feitsui artificial water reservoir (Taiwan; accession numbers: AB930693.1 & AB930564.1) [34]. Additionally, the Lugano sequence clustered together with sequences retrieved from bioreactors inoculated from wastewater treatment plants. This cluster formed a distinct clade within the *Ca*. Azoamicus genus (Fig. 3A). The MAG of the Lake Lugano endosymbiont showed an overall 99.6% ANI to the MAG of *Ca*. A. ciliaticola (GCF_902860225.1) [5] and thus represents a genomovar (99.6%–99.98% ANI [35]) of *Ca*. A. ciliaticola. We will further refer to these genomovars as *Ca*. A. ciliaticola Zug and *Ca*. A. ciliaticola Lugano, respectively. The two MAGs were very similar in terms of size (ca. 290 kb) and GC content (24.4%) and encoded the same number of total genes (348), showing conserved synteny, with the exception of one hypothetical gene missing in *Ca*. A. ciliaticola Lugano. Thus, the Lake Lugano endosymbiont also encoded all genes that would enable *Ca*. A. ciliaticola Zug to function as an obligate respiratory endosymbiont, including genes for respiratory nitrate reduction (*narGHIJ,* nirK, *norBC, nosZ*) and energy conservation (ATP synthase, *atpA – atpI*; ATP/ADP translocase, *tlcA*).

A survey of nine additional amplicon datasets collected between 2015 and 2018 from Lake Lugano's permanently stratified northern basin [12] provided further insights into the diversity of *Ca*. Azoamicus endosymbionts in Lake Lugano. Eight OTUs that shared 93.1%–100% identity to the 16S rRNA gene of *Ca*. A. ciliaticola Lugano were detected (Fig. 3A). Of those, five formed a distinct monophyletic clade together with *Ca*. A. ciliaticola Lugano (97.3%–100% identity). Three additional, more distantly related OTUs (93.1%–93.3% identity) grouped together with *Ca*. Azoamicus parvus, which is commonly found in wastewater treatment plants around the world [7]. All eight OTUs detected in Lake Lugano fall into clades within the *Ca*. Azoamicus genus, which seems to have secondarily lost the genes encoding the cytochrome-*cbb_3_* oxidase [7]. Indeed, genes encoding for terminal oxidase were not found in *Ca*. A. ciliaticola Lugano genome generated in this study. Additional screening of the Lake Lugano metagenomes from 90, 100, and 130 m depth for reads of the *ccoN* gene recovered only two reads from 100 m depth (see Supplementary Methods). This means that the cytochrome-*cbb_3_* oxidase gene is absent from these metagenomic datasets.

### Phylogeny of plagiopylids from Lake Lugano and their distribution in the water column

The distribution of *Ca*. A. ciliaticola Lugano endosymbionts and their hosts in the water column in August 2020 was investigated using FISH. The endosymbionts were identified using the same oligonucleotide probe as in Lake Zug (eub62A3_813). The probe is predicted to target five of the eight OTUs identified in Lake Lugano amplicons, which were the most abundant OTUs in 72% of all samples in which endosymbionts were detected (Table S2). FISH analyses of water samples from 75 to 250 m depth showed that, when *Ca*. A. ciliaticola Lugano was present, it was exclusively detected inside a eukaryotic host. The host was identified as a ciliate, primarily based on the presence of both a macro- and micronucleus. Additionally, the ciliate displayed an overall high morphological similarity—regarding size, shape, and autofluorescence—to the plagiopylid host of *Ca*. A. ciliaticola in Lake Zug. Double hybridization with a Plagiopylea-specific oligonucleotide probe (plagi_1083) [7], together with the endosymbiont-specific eub62A3_813 probe, was performed on samples retrieved from 95 and 100 m depth, in which the presence of *Ca*. A. ciliaticola was corroborated. Double hybridization confirmed that the *Ca*. A. ciliaticola Lugano host was a plagiopylean ciliate (Fig. 2C). The host plagiopylids were only detected at depths between 85 and 110 m, reaching the highest abundance of ca. 8400 plagiopylids L^−1^ at 95 m depth (Fig. 2B). All ciliates hybridized with the plagi_1083 probe also exhibited *Ca*. Azoamicus-specific fluorescence signals (see also Fig. S9).

The depth distribution of the five symbiont OTUs belonging to the *Ca*. A. ciliaticola clade showed distribution patterns that mirrored the FISH-based ciliate counts. The three OTUs belonging to the *Ca*. A. parvus clade did not exhibit a clear depth pattern, possibly due to their generally lower abundance. Overall, OTUs belonging to the *Ca*. Azoamicus genus, with a minimum relative abundance of 0.01%, were detected in all analyzed campaigns in Lake Lugano, in waters with oxygen concentrations ranging from 0 to 33 μM and sulfide concentrations from 0 to 4.9 μM. However, all OTUs had the highest abundances in the water layers between the oxic–anoxic interface and the deeper sulfidic layers (Fig. S4, Table S2).

We were unable to retrieve an 18S rRNA gene sequence of the plagiopylean host from Lake Zug (LR798089 [5]) in the Lake Lugano metagenome using phyloFlash v. 3.3 [36]. Therefore, we designed specific 18S rRNA gene primers targeting the class Plagiopylea, in order to amplify the host 18S rRNA gene from the water samples (90, 100, and 130 m). We could successfully amplify two ca. 800 bp-long 18S rRNA gene fragments, one from 100 m and one from 130 m depth. These two retrieved 18S rRNA gene sequences showed high similarity to each other (99.6% identity), as well as to the 18S rRNA gene sequence of the Lake Zug host (99.6%–100% identity). Using only the forward amplicon reads from Lake Lugano, we were able to get a broader picture of the diversity of potential host ciliates in Lake Lugano. Seven OTUs that shared 89.7%–100% identity over the full amplicon sequence (270 bp), to the plagiopylean host from Lake Zug were detected (Fig. 3B). Of those, four formed a distinct monophyletic clade together with the plagiopylean host from Lake Zug (94.8%–100% identity). Three additional, more distantly related OTUs (89.7%–93% identity) grouped together with other environmental sequences from marine sediments and wastewater. The two clades are well supported; their exact position in the tree and the phylogenetic relation to each other could, however, not be resolved conclusively.

### *In situ* growth and division of the anaerobic ciliate host

**Figure 4:**
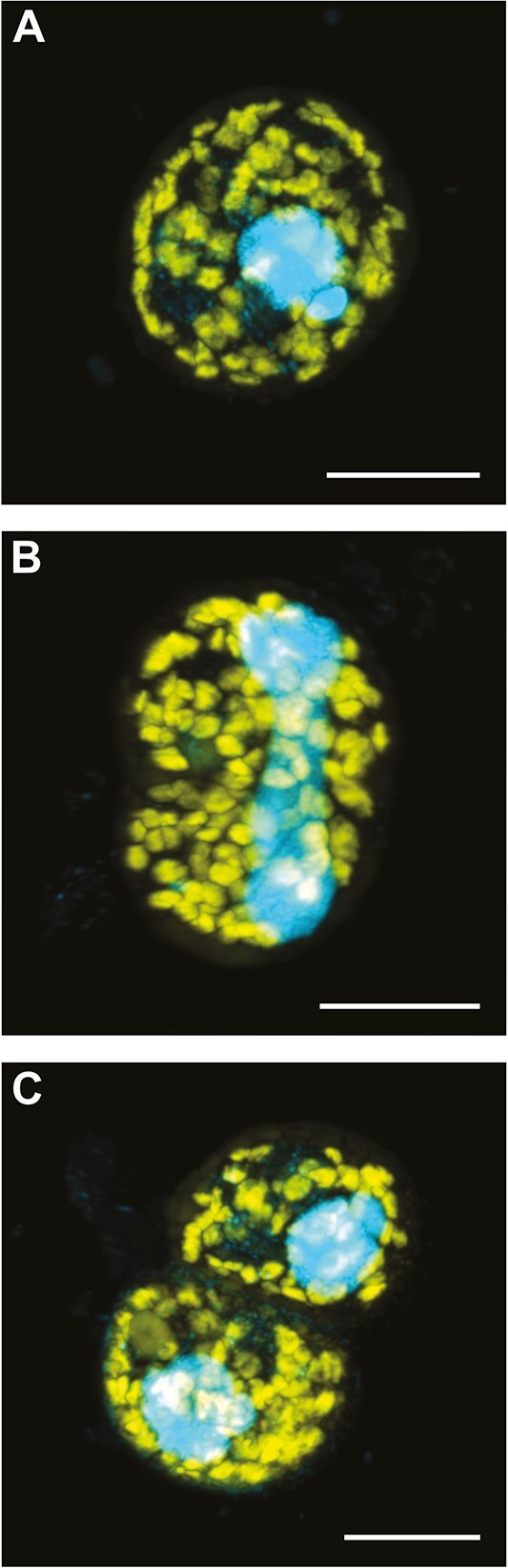
Different stages of cell division in Lake Zug plagiopylids and the distribution of their endosymbionts. The different stages of ciliate division comprise (A) the vegetative stage, (B) elongation of macro- and micronucleus, and (C) division of macro- and micronucleus with final cell division. Plagiopylid nuclei were counterstained with DAPI (blue). Endosymbionts were visualized with the *Ca*. Azoamicus–specific probe eub62A3_813 (yellow). Image stacks were recorded with a confocal laser scanning microscope and represent maximum intensity projections. Images were recorded from samples collected from Lake Zug 160 m water depth in July 2023. Scale bars, 10 μm.

During the microscopy screening of the water samples from Lake Zug and Lake Lugano, we frequently encountered apparently dividing plagiopylids. In Lake Zug, various stages of division were detected in approximately one-third of all investigated depths over all campaigns, except November 2021. The majority of dividing plagiopylids was encountered in samples from anoxic water depths from 140 to 190 m depth. Only in October 2019, dividing plagiopylids were detected in waters containing up to 10 μM oxygen. Based on FISH counts (Table S3), on average, ~1% of the plagiopylid population was in the process of division, with the highest proportion typically found in anoxic depths with the highest overall ciliate abundance. In Lake Zug, the highest proportion of dividing ciliates (6.5%) was recorded in July 2019 at a depth of 185 m, just below the depth with the highest ciliate abundance. Dividing ciliates were also observed in Lake Lugano, with 2.3% and 8% of the plagiopylids undergoing division at the anoxic depths of 90 and 95 m, respectively. The individual division stages comprised the initial furrow formation of the cell body and the elongation of both the macro- and micronucleus (Fig. 4B), followed by the continued elongation of both nuclei, and subsequently their distribution into the two daughter cells (Fig. 4C).

Due to the high numbers of dividing plagiopylids, we could investigate the distribution of *Ca*. A. ciliaticola symbionts in their hosts during multiple stages of asexual division using FISH. In nondividing plagiopylids, endosymbiont signals were dispersed evenly across the host cell, often near the periphery, or formed aggregates closer to the center (Figs 4A and S8), as observed before [5]. During division, endosymbionts were always present, and, in most cases, appeared to be evenly dispersed across the two forming daughter cells (Fig. 4). Upon division, both daughter cells received comparable amounts of endosymbiont cells (average 40 and 47, *n* = 5; plagiopylids from Lake Zug). This is consistent with the average of 44 endosymbionts per host cell in nondividing plagiopylids (*n* = 60; plagiopylids from Lake Zug).

## Discussion

### Diversity of *Ca*. Azoamicaceae in Lake Lugano

Despite freshwater being the most common habitat for respiratory endosymbionts [6], their diversity, as well as the identity of their hosts, remains understudied. Yet, this knowledge is crucial in order to understand the evolutionary history and ecological importance of this symbiosis.

Amplicon analysis of Lake Lugano revealed multiple OTUs from the *Ca*. Azoamicus genus, sharing between 93% and 100% sequence identity with the 16S rRNA gene sequence of *Ca*. A. ciliaticola Lugano. The most abundant OTUs, including an OTU identical to the 16S rRNA sequence of *Ca*. A. ciliaticola Lugano, form a monophyletic clade with the 16S rRNA sequence of *Ca*. A. ciliaticola Zug. The closed genome of *Ca*. A. ciliaticola Lugano is 99.6% identical to the one from Lake Zug in terms of sequence identity and gene content, differing only by the absence of one hypothetical gene in *Ca*. A. ciliaticola Lugano. Other, less abundant OTUs retrieved from Lake Lugano were more closely related (94%–95% identity) to a different species, *Ca. A. parvus*. This species has the smallest genome of all described *Ca*. Azoamicaceae symbionts and is widespread in wastewater treatment plants around the world [7]. In general, the diversity of *Ca*. Azoamicaceae in wastewater treatment plants is higher than in the natural habitats, such as groundwater and freshwater lakes [6, 7]. However, the freshwater symbiont diversity is more constrained, even compared to groundwater, from which two distinct genera of *Ca*. Azoamicaceae are known [6].

### *Ca*. A. ciliaticola is host specific and vertically transmitted

The ciliates harboring *Ca*. A. ciliaticola in Lake Lugano looked morphologically indistinguishable from the plagiopylid ciliates from Lake Zug. Double labelling of the hosts and symbionts with specific 16S and 18S rRNA FISH-probes has unambiguously shown that *Ca*. A. ciliaticola Lugano also associates with a plagiopylean ciliate. Correspondingly, 18S rRNA gene amplification with Plagiopylea-specific primers recovered two partial 18S rRNA gene sequences, which were almost identical (>99.5% identity) to the plagiopylean host from Lake Zug. These data show that *Ca*. A. ciliaticola Lugano associates with the same ciliate host as *Ca*. A. ciliaticola Zug. Based on the available evidence, we thus speculate that *Ca*. A. ciliaticola, which is by far the most common species in global amplicon data [6], might always associate with the same plagiopylean host.

*Candidatus* Azoamicaceae species retrieved from other environments (e.g. wastewater and groundwater) have also been shown to coexist with plagiopylean ciliates [6, 7]. Indeed, the overall relative abundance of plagiopylean OTUs correlates significantly with the overall relative abundance of *Ca*. Azoamicaceae OTUs in Lake Lugano (see Table S2). This suggests that also other detected Ca. Azoamicaceae, presumably related to *Ca*. A. parvus, might associate with plagiopylean ciliates. We did retrieve three plagiopylean OTUs (OTU 5, 6, and 7) that clustered in a separate clade and were most closely related to sequences retrieved from marine and wastewater samples. Thus, there might be other, albeit less abundant, plagiopylid ciliate species with respiratory endosymbionts present in Lake Lugano.

Microscopic observations of the water samples from Lake Lugano, as well as Lake Zug, revealed the presence of dividing ciliates. Ciliates reproduce both asexually by division and sexually through conjugation [1]. Sexual reproduction is thought to be much rarer but could represent a potentially important mechanism for endosymbiont dispersion, as during conjugation, micronuclei, as well as in some cases, parts of the cytoplasm of the two partners (e.g. including their mitochondria), are exchanged [37]. It is not clear whether conjugation could also lead to an exchange of endosymbiont populations, and unfortunately, conjugation was not observed in our investigated lake ciliate populations. Ciliates are also known to form cysts when environmental conditions are unfavorable, and some anaerobic ciliates sequester their endosymbionts into these cysts [38]. At the moment, there are no reports of encystment in Plagiopylea, but it is a potential mechanism for the dispersal of this symbiosis.

On average, 1% of the ciliate population were found to be dividing in the lake samples and all relevant stages of ciliate division were encountered. The endosymbionts were evenly distributed into the daughter cells and the number of symbiont cells in the newly formed daughter cells was very similar to the number of endosymbionts in vegetative host cells. These results show that during the asexual cell division, *Ca*. A. ciliaticola is vertically transmitted. Moreover, this suggests that the symbiont population only divides prior to or simultaneously with the host, suggesting a tightly regulated symbiosis. It has been shown for the related species *Plagiopyla frontata* that its methanogenic endosymbiont divides in synchrony with the host ciliate’s cell cycle [3]. The transmission of endosymbionts to the next host generation is a crucial step for the maintenance of long-term symbiosis [39]. However, the symbioses between anaerobic ciliates and their methanogenic endosymbionts are often stable only over short time scales [40–42]. This is in contrast to the respiratory symbionts, whose strongly reduced genome suggests a long-term intracellular lifestyle without the capacity to survive outside their host. Nonetheless, more endosymbiont species will have to be linked to a specific host species, to unambiguously demonstrate a long-term co-evolution of plagiopylean ciliates and *Ca*. Azoamicaceae endosymbionts.

### Lacustrine plagiopylids niche is determined by redox conditions

The abundances of plagiopylids in Lake Zug varied during the 10 sampling campaigns spanning six consecutive years. Although their cell counts remained generally low (ca. 1800 l^−1^), in some years these increased to ca. 10 000–20 000 l^−1^, with the highest abundance found in July 2021 (ca. 35 000 l^−1^). The reasons for these fluctuations remain unclear; however, clear trends in ciliates’ depth distribution in the water column were observed.

The highest abundance of plagiopylids was consistently observed in anoxic, nitrate-replete waters. This is consistent with their obligately anaerobic lifestyle and reliance on nitrate as the electron acceptor for denitrification [5]. Based on these and previous observations, oxygen depleted, nitrate-rich freshwater bodies emerge as a common habitat for the obligately anaerobic plagiopylids harboring *Ca*. Azoamicus endosymbionts. It should be pointed out that plagiopylids with the facultatively anaerobic *Ca*. Azoamicus symbionts, which encode a cytochrome*-cbb_3_* oxidase, are also prevalent in nitrate-rich wastewater treatment plants [7] and relatives of the *Ca*. Azosocius genus have been retrieved from nitrate-containing groundwater of varying oxygen concentrations [6].

The vertical distribution of the plagiopylids in the water column of Lake Lugano showed that they were virtually absent from euxinic waters. Even though it is possible that this is due to the absence of nitrate in these waters, a toxic effect of sulfide on the host or symbiont cannot be excluded, as in our laboratory test plagiopylids died within minutes after exposure to 10 μM sulfide (see Material and Methods). Although sulfide is notoriously toxic for a range of aquatic organisms, such as plants and animals [43], anaerobic ciliates, even freshwater ones, are known to tolerate often high sulfide concentrations (up to 700 µM [44]). For example, a closely related ciliate, *Plagiopyla nasuta,* survived exposure to up to 4.5 mM sulfide [45] and anaerobic ciliates have been reported to thrive in sulfidic marine sediments [3, 46, 47]. Sulfide toxicity in eukaryotes is often attributed to the inhibition of complex IV of the respiratory chain, which is not present in *Ca*. A. ciliaticola nor its host. Also, the formation of reactive oxygen species has been linked to sulfide toxicity [48, 49], which seems, however, an unlikely scenario in anoxic lake waters. It was further described that sulfide can reduce cytochrome *c* [49]. Whereas cytochromes are not present in anaerobic ciliates harboring methanogenic endosymbionts [3], they are the electron transporters in the electron transport chain of *Ca*. A. ciliaticola and its relatives [5, 6]. Reduction of cytochrome *c* would interrupt the electron transport chain of *Ca*. A. ciliaticola, which might provide an explanation for the apparent sulfide sensitivity of these anaerobic ciliates.

Also, in marine oxygen minimum zones, protists were shown to follow biogeochemical gradients such as of oxygen or sulfide [50–52]. As many anoxic marine sediments show elevated sulfide concentrations [53], we speculate that these might not be a suitable habitat for these plagiopylean ciliates with denitrifying endosymbionts. However, presumably favorable conditions can be found in the water column of marine oxygen minimum zones that are oxygen-deficient but nitrate-replete. Not only do these host free-living denitrifying bacteria [54] but also eukaryote-associated denitrification was suggested to play an important role [55, 56].

Typically, the abundance of prey (bacteria or flagellates) is a major factor affecting the distribution of heterotrophic ciliates in the water column of meromictic lakes [57, 58]. In Lake Zug, the plagiopylid abundances did not show statistically significant correlation with bacterial or small protist cell counts in their respective depths, even though the lowest abundance of small protists coincided with the highest plagiopylid abundance in May 2022 (160 m depth). Sequences belonging to Cryptomonadales flagellates were retrieved during sequencing of single plagiopylid cells from Lake Zug earlier [5], suggesting that *in situ*, both bacteria and small eukaryotes may constitute part of the plagiopylid diet. Thus, although the exact nature of their prey remains unclear, these plagiopylids may also be predators. In contrast, anaerobic ciliates, including the members of the major Plagiopylea clades Trimyemidae, Plagiopylidae, and Sonderiidae, are typically bacterivorous [3].

Overall, our data suggest that in Lake Zug and Lake Lugano, redox conditions dictated by the gradients of oxygen and nitrate are the major factors controlling the vertical distribution of these plagiopylid ciliates (Figs 1 and 2). The notable upward shift of the plagiopylids into shallower, but oxygen-depleted waters observed during the November 2021 sampling campaign, and onwards, coincided with the depletion of nitrate in deeper waters. The plagiopylids abundances were consistently highest at depths at which nitrate was nearly depleted. The presumable bloom of these organisms in July 2021, with exceptionally high abundances of ca. 35 000 cells l^−1^ at 180 m coincided with a complete removal of nitrate at this depth (Fig. 1). Indeed, at this high abundance the plagiopylids alone were modeled to remove about half of the nitrate that diffuses into this depth (see Material and Methods and Table S5). These observations suggest that denitrifying plagiopylids at times substantially contribute to nitrate consumption in the water column of Lake Zug. It has been suggested previously that other denitrifying eukaryotes, such as the ciliate *Loxodes* and the foraminifera *Nonionella* sp., can also substantially contribute to nitrate removal from lakes and coastal sediments, respectively [59, 60]. Together, these observations reinforce the emerging role of eukaryotes in aquatic nitrogen loss.

## Supplementary Material

wrag043_Supplements_Zeller_Schorn_et_al

wrag043_Supplementary_materials

## Data Availability

The Lake Lugano metagenomes and the MAG of *Ca*. A. ciliaticola Lugano were deposited at NCBI under the BioProject PRJNA1294207. The amplicon data can be found at NCBI under the BioProject PRJNA772618. The specific accession numbers of the amplicon data are listed in Table S4.
