# Supplementary material for: Redox gradients define the ecological niche of ciliates with denitrifying endosymbionts in anoxic lake waters
Source: ISME J. 2026 Mar 1;20(1):wrag043. doi: 10.1093/ismejo/wrag043 (PMC13037464; doi:10.1093/ismejo/wrag043)
Supplement: wrag043_Supplementary_materials [file wrag043_supplementary_materials.zip › Supplements_Zeller_Schorn_et_al.docx]

**Supplementary Material**

**Redox gradients define the ecological niche of ciliates with denitrifying endosymbionts in anoxic lake waters**

Linus M. Zeller^1^*, Sina Schorn^1,2^*, Louison Nicolas-Asselineau^1^, Jakob Zopfi^3^, Soeren Ahmerkamp^1,4^, Carsten J. Schubert^5,6^, Fabio Lepori^7^, Marcel M. M. Kuypers^1^, Jon S. Graf^1^, and Jana Milucka^1^

* these authors contributed equally

1 Max Planck Institute for Marine Microbiology, Bremen, Germany

2 Department of Marine Sciences, University of Gothenburg, Gothenburg, Sweden

3 Department of Environmental Sciences, University of Basel, Basel, Switzerland

4 Leibniz Institute for Baltic Sea Research, Rostock, Germany

5 Department of Surface Waters-Research and Management, Swiss Federal Institute of Aquatic Science and Technology (EAWAG), Kastanienbaum, Switzerland

6 Institute of Biogeochemistry and Pollutant Dynamics, ETH Zurich, Zurich, Switzerland

7 Institute of Earth Sciences, University of Applied Sciences and Arts of Southern Switzerland (SUPSI), Mendrisio, Switzerland

**Corresponding author:** Linus M. Zeller, [lzeller@mpi-bremen.de](mailto:lzeller@mpi-bremen.de)

**Mailing address:** Max-Planck-Institute for Marine Microbiology, Celsiusstraße 1, 28359 Bremen, Germany

**Table of content**

[Methods 2](#_Toc222401941)

[Double hybridization of host and endosymbiont using FISH 2](#_Toc222401942)

[Enumeration of small protists 3](#_Toc222401943)

[Gradient PCR 3](#_Toc222401944)

[18S rRNA amplicon analysis 4](#_Toc222401945)

[Small subunit rRNA gene phylogeny 4](#_Toc222401946)

[Search for the *ccoN* gene in Lake Lugano metagenomes 5](#_Toc222401947)

[Calculation of ciliate and endosymbiont cell volumes 5](#_Toc222401948)

[Supplementary Results 5](#_Toc222401949)

[Identification of protists in the water column 5](#_Toc222401950)

[Specificity of plagiopylean 18S rRNA gene primers 6](#_Toc222401951)

[References 6](#_Toc222401952)

[Supplementary figure text 8](#_Toc222401953)

# Methods

## Double hybridization of host and endosymbiont using FISH

Double hybridization of host and endosymbiont was conducted using FISH as described previously ^1^. Filter pieces were embedded in 0.2% MetaPhor Agarose (Lonza), and hybridized with oligonucleotide probe plagi_1083 (terminally double-labelled with Atto594 dye) for 2 h at 46°C at 35% formamide concentration, then washed for 30 minutes in washing buffer (0.7 mL 5 M NaCl, 1 mL 1M Tris*HCl pH 8, 0.5 mL 0.5 M EDTA pH 8, filled up to 50 mL with milliQ water) at 48°C, and 10 minutes in PBS at room temperature. The same filters were then hybridized with probe eub62A3_813 (terminally double-labelled with Atto488 dye) for 3 to 5 h at 25% formamide concentration, and washed as described above. Filters were stained with 1 µg mL^-1^ 4′,6-diamidino-2-phenylindole (DAPI) for 10 minutes at 4°C. As a control, the probe Non488 (5’-ACTCCTACGGGAGGCAGC-3') was used on separate filter pieces (**Fig. S9**).

## Enumeration of small protists

Small protists were enumerated in samples collected from Lake Zug in May 2022 by FISH as described previously ^2^. Filter pieces were embedded in 0.2% MetaPhor Agarose (Lonza) followed by permeabilization with 15 µg mL^-1^ proteinase K for 10 minutes at room temperature. Filters were washed in phosphate-buffered saline (PBS), and endogenous peroxidases were inactivated in 0.01 M hydrochloric acid for 10 minutes. The filter pieces were hybridized with the four eukaryote-specific horseradish peroxidase-labelled probes: EUK 1209 (5'-GGGCATCACAGACCTG-3'), EUK 502 (5'-ACCAGACTTGCCCTCC-3'), EUK 309 (5'-TCAGGCTCCCTCTCCGG-3'), and EUK B (5'-TGATCCTTCTGCAGGTTCACCTAC-3') ^3^ as well as the competitor probe KIN 516 (5’ -ACCAGACTTGTCCTCC-3') ^4^. Probes were mixed in equal amounts in 40% formamide hybridization buffer, and incubated for 3 hours at 46°C. Afterwards, filters were washed 5 minutes in preheated washing buffer (0.46 mL 5 M NaCl, 1 mL 1M Tris*HCl pH 8, 0.5 mL 0.5 M EDTA pH 8, filled up to 50 mL with milliQ water) at 48°C followed by 15 minutes in 1x PBS at room temperature. Amplification was performed with OregonGreen488-labeled tyramides at 46°C for 25 minutes. Filters were then washed in preheated PBS at 48°C, followed by briefly rinsing in milliQ water, and absolute ethanol. Filter pieces were DAPI counterstained, and embedded for microscopy as described in Fluorescence *in situ* hybridization (**Material and Methods**). Cells with a positive FISH signal were counted in at least 20 fields of view using a counting grid (125×125 µm).

## Gradient PCR

The specific annealing temperature of the primer pair plagi_289_F (5’-TCAAGTTTCTGCCCTATCAC-3’) and plagi_1107_R (5’-TCAGACTTGTGTCCATACTT-3’) was experimentally tested at a temperature gradient from 48°C to 63°C. DNA extracted from Lake Zug water collected from 185 m depth (November 2021) was used as a template. The PCR amplifications (25 µL) were performed with 0.4 ng µL^-1^ template, 2.5 µL 10 × PCR buffer (ThermoPol, NEB), 2 µl dNTP’s (40 mM) (Roche), 0.25 µL of each primer (50 pmol µL^-1^), 2.5 µL 3 µg µL^-1^ BSA and 0.1 µL 5 U µL^-1^ Taq-polymerase (NEB). The following PCR scheme was applied for 30 cycles in the Biometra TAdvanced (Analytic Jena): initial denaturation for 2 minutes at 94°C, denaturation for 30 s at 94°C, annealing for 30 s at a temperature gradient of 46°C to 64°C, amplification for 70 s at 72°C, final amplification for 10 minutes at 72°C. DNA was visualized on a 1% agarose gel (Biozym LE Agarose) using SYBR Safe DNA stain (Invitrogen) on a UVP ChemStudio PLUS (Analytik Jena). A single PCR product of about 800 bp was amplified from 48°C to 63°C. At temperatures above 58°C the intensity of the PCR band visibly decreased (**Fig. S1**). For all the following PCRs the primer pair was used at an annealing temperature of 58°C.

**Sanger Sequencing**

The 18S rRNA gene PCR products from Lake Lugano were prepared for Sanger sequencing as described previously ^5^. Briefly, the PCR products were purified using gel filtration (Sephadex G-50 Superfine, Amersham Bioscience), followed by amplification using the forward and reverse primers with the BigDye Terminator v3.1 Cycle Sequencing Kit (Thermo Fisher Scientific) and sequenced in the automated DNA capillary sequencer (3130XL Genetic Analyzer, Applied Biosystems).

## 18S rRNA amplicon analysis

The forward reads of the amplicon sequences were downloaded using fasterq-dump v. 2.11.0 (<https://github.com/ncbi/sra-tools>) and imported as single-end reads into QIIME 2 v. 2022.11.1 ^6^. The reads were trimmed using cutadapt ^7^ to remove the 515F-Y (5′-GTGYCAGCMGCCGCGGTAA-3’) adapters. Denoising was conducted using DADA2 ^8^. In order to keep a Phred score generally above 20 the reads were truncated after 270 bp. Representative sequences were clustered to reduce redundancy into operational taxonomic units (OTUs) with a minimum of 99% identity with VSEARCH ^9^. OTUs were finally blasted against the 18S rRNA gene of the plagiopylean host from Lake Zug using blastn ^10,11^.

## Small subunit rRNA gene phylogeny

For the 16S rRNA gene phylogeny, the 16S rRNA gene of the Lake Lugano MAG was used together with the 16S rRNA gene sequences of *Ca.* Azoamicaceae obtained from the amplicon analysis. Reference sequences were obtained from ^1^ together with the closest related 16S rRNA gene sequences from NCBI, originating from lakes. As an outgroup five randomly selected sequences from the sister group of the order Legionella were taken from the European Nucleotide Archive (<https://www.ebi.ac.uk/ena/browser/home>) (**Supplementary File 6**).

For the 18S rRNA gene phylogeny, partial sequences retrieved from Sanger sequencing were used together with plagiopylean sequences retrieved from the amplicon analysis. Reference sequences were taken from ^1^, and as an outgroup three randomly selected 18S rRNA gene sequences from Plagiopylida were taken from NCBI (**Supplementary File 7**).

## Search for the *ccoN* gene in Lake Lugano metagenomes

The operon coding for a cytochrome-*cbb3* oxidase of Azoamicaceae origin was searched for in the metagenomes collected from depths 90 m, 100 m, and 130 m in Lake Lugano in 2020. The trimmed metagenomic reads were mapped on the *ccoN* gene sequences found in previously recovered respiratory endosymbiont genomes ^1,12^ with an identity of 95% over a minimum of 80% of the read using CoverM 0.6.1 (<https://github.com/wwood/CoverM>). The *ccoN* gene was chosen as a reference as it codes for a catalytic subunit of the cytochrome-*cbb3* oxidase ^13^.

## Calculation of ciliate and endosymbiont cell volumes

Ciliate cell volumes (V_c_) were calculated based on the measured length, and width of 60 ciliates, assuming a prolate spheroid cell shape. Accordingly, the volume was calculated using the formula:

$V_{c}= \frac{\pi}{6}*{width}^{2}*length$

In addition to measuring the size, and volume of each ciliate, we also quantified the number of endosymbionts per cell (V_e_), as well as their intracellular distribution, size, and volume. The volume of endosymbiont cells was calculated using the formula:

$V_{e}=\frac{4}{3}*\pi*{r_{e}}^{3}$

where r_e_ is the radius of the spheroid bacterial cell. We observed distinct patterns of endosymbiont distribution within the ciliate hosts, which we categorized based on their location as follows: aggregated, referring to large clusters of endosymbionts near the cell center; peripheral, where endosymbionts were positioned close to the cell’s periphery; and dispersed, indicating an even spread of endosymbionts throughout the cell. In total, we counted and categorized > 2500 endosymbionts (**Tab. S3**).

# Supplementary Results

## Identification of protists in the water column

Ciliates without *Ca.* A. ciliaticola endosymbionts (identified based on the presence of macro- and micro-nucleus, but the absence of an endosymbiont FISH signal) were only sporadically observed in anoxic depths, but they were more common at the oxic-anoxic interface. These ciliates were often larger, had a more elongated cell shape, and did not exhibit the characteristic autofluorescence of *Ca*. A. ciliaticola hosts. At anoxic depths, smaller protists, reminiscent of flagellates, and diatoms, were also present (**Fig. S2**). Thus, *Ca.* A. ciliaticola-hosting plagiopylids could be clearly differentiated from other protists in anoxic waters.

## Specificity of plagiopylean 18S rRNA gene primers

The forward primer plagi_289_F (5’-TCAAGTTTCTGCCCTATCAC-3’) (number indicating the 5’- binding position on the 18S rRNA gene sequence of the plagiopylean host of *Ca.* A. ciliaticola (NCBI accession number LR798089.1)), binds to all sequences within the Plagiopylea genus, with the exception of one *Trimyema* sequence. It also binds to 4 unrelated sequences (one uncultured Chrysophyte, one uncultured Ochromonas, one uncultured Cercozoan, and *Navicula phyllepta*)*.* With one accepted mismatch, the primer binds to 3813 sequences. With a few, exceptions all these mismatches are at the 3’-end of the sequence. The reverse primer plagi_1107_R (5’-TCAGACTTGTGTCCATACTT-3’) binds only to sequences within the Plagiopylea, with a mismatch to the 13 sequences of the genus *Trimyema*. With one accepted mismatch the primer binds to 90 sequences. Among those are the *Trimyema* sequences, with a weak mismatch (G instead of A) in the middle of the primer. The other species can be found within the Mesodiniidae, with a mismatch at the 3’-end of the primer sequence. Because of the high specificity of the reverse primer, and because most mismatches are at the 3’-end of the primers, we assumed that this primer pair is highly specific to the class Plagiopylea.

# References

1. Nicolas-Asselineau, L., Speth, D. R., Zeller, L.M. *et al.* Occurrence and temporal dynamics of denitrifying protist endosymbionts in the wastewater microbiome. *ISME Commu.* **5**, ycaf209 (2025).

2. Pernthaler, Annelie & Pernthaler, J. Fluorescence in situ hybridization for the identification of environmental microbes. *Protocols for Nucleic Acid Analysis by Nonradioactive probes* **353**, 153–164 (2007).

3. Lim, E. L., Caron, D. A. & Delong, E. F. Development and field application of a quantitative method for examining natural assemblages of protists with oligonucleotide probes. *Appl. Environ. Microbio.l* **62**, 1416–1423 (1996).

4. Bochdansky, A. B. & Huang, L. Re‐evaluation of the EUK516 probe for the domain eukarya results in a suitable probe for the detection of Kinetoplastids, an important Group of Parasitic and Free‐Living Flagellates. *J. Eukaryotic Microbiology* **57**, 229–235 (2010).

5. Bondoso, J., Harder, J. & Lage, O. M. rpoB gene as a novel molecular marker to infer phylogeny in Planctomycetales. *Antonie van Leeuwenhoek* **104**, 477–488 (2013).

6. Bolyen, E., Rideout, J. R., Dillon, M. R. *et al.* Reproducible, interactive, scalable and extensible microbiome data science using QIIME 2. *Nat. Biotechnol.* **37**, 852–857 (2019).

7. Martin, M. Cutadapt removes adapter sequences from high-throughput sequencing reads. *EMBnet J.* **17**, 10 (2011).

8. Callahan, B. J., McMurdie, P. J., Rosen, M. J. *et al.* DADA2: high-resolution sample inference from Illumina amplicon data. *Nat. Methods* **13**, 581–583 (2016).

9. Rognes, T., Flouri, T., Nichols *et al*. VSEARCH: a versatile open source tool for metagenomics. *PeerJ* **4**, e2584 (2016).

10. Altschul, S. F., Gish, W., Miller, W. *et al*. J. Basic local alignment search tool. *J. of Molecular Biology* **215**, 403–410 (1990).

11. Camacho, C., Coulouris, G, Avagyan, V *et al.* BLAST+: architecture and applications. *BMC Bioinformatics* **10**, (2009).

12. Speth, D. R., Zeller, L. M., Graf, J. S. *et al.* Genetic potential for aerobic respiration and denitrification in globally distributed respiratory endosymbionts. *Nat. Commun.* **15**, 9682 (2024).

13. Thöny‐Meyer, L., Beck, C., Preisig, O. *et al*. The *ccoNOQP* gene cluster codes for a *cb* ‐type cytochrome oxidase that functions in aerobic respiration of *Rhodobacter capsulatus*. *Molecular Microbiology* **14**, 705–716 (1994).

# Supplementary figure text

**Figure S1 Gradient PCR of the primer pair plagi_289_F and plagi_1107_R, designed to target the 18S rRNA gene of plagiopylean ciliates. The primer pair amplifies a ca. 800 bp long DNA fragment. The annealing temperature is given at the top of the respective column in degrees Celsius (°C). The primer pair was further used with an annealing temperature of 58°C. L = DNA ladder (100 bp Plus, GeneRuler).**

**Figure S2 Protists other than the plagiopylean ciliates detected in the anoxic waters of Lake Zug. A. flagellates (stained with DAPI, blue) and B. diatoms (displaying autofluorescence at 488 nm, green). Scale bars, 10 µm.**

**Figure S3 Abundance of A. plagiopylids (yellow bars), B. protists (smaller than 20 µm; magenta bars) and C. prokaryotes (blue bars) in Lake Zug in May 2022. Counts were done on PFA-fixed samples on filters, as described in Material and Methods and Supplementary Methods. Plagiopylids were counted using the endosymbiont-specific FISH-probe eub62A3_813, small protists were counted using FISH-probes specific for eukaryotes (EUK 1209, EUK 502, EUK 309 and EUK B) and prokaryotes were counted after DAPI staining.**

**Figure S4 Oxygen (grey line), nitrate (blue dots) and sulfide (red dots) concentrations and the relative abundance of *Ca.* Azoamicaceae operational taxonomic units (OTUs) in the anoxic hypolimnion of Lake Lugano. Three of the nine campaigns, in which amplicon data were collected, are displayed (April 2015, June 2015, September 2016). Redox conditions, as well as the respective sampling date, are depicted in the left panel (A, D, G). The relative abundances of the OTUs forming a clade with *Ca.* A. ciliaticola (ZOTU13681, ZOTU2307, ZOTU1067, ZOTU1467, ZOTU7928) were summed up and are shown in the middle panel (B, E, H). The relative abundances of the OTUs forming a clade with *Ca.* A. parvus (ZOTU5612, ZOTU7194, ZOTU32) were summed up and are shown in the right panel (C, F, I). The data for all campaigns can be found in Table S2.**

**Figure S5 16S rRNA gene sequence-based maximum likelihood phylogenetic tree of *Ca*. Azoamicaceae. Sequences were retrieved from Lake Lugano metagenomes and amplicon sequences. Additional members include related gammaproteobacterial orders and environmental clades. Bootstrap support of >90% (1000 resamplings) are shown as black circles at the respective nodes. Scale bar indicate 0.05 nucleotide substitutions per site.**

**Figure S6 Ciliate 18S rRNA gene sequence-based maximum likelihood phylogenetic tree of the class Plagiopylea. Sequences of ciliates from Lake Lugano were amplified from samples collected from 100 m and 130 m by polymerase chain reaction using a Plagiopylea-specific primer pair. Bootstrap values are shown as circles at the respective nodes and indicate bootstrap support of >80% (grey) or >90% (black) out of 1000 resamplings. Scale bar indicates 0.05 nucleotide substitutions per site.**

**Figure S7 Ciliate 18S rRNA gene sequence-based maximum likelihood phylogenetic tree of the class Plagiopylea. Sequences of ciliates from Lake Lugano were amplified from samples collected from 100 m and 130 m by polymerase chain reaction using a plagiopylea-specific primer pair. Bootstrap values are shown as black circles at the respective nodes and indicate bootstrap support >90% out of 1000 resamplings. Scale bar indicates 0.05 nucleotide substitutions per site. This tree is a version of the tree shown in Fig. 3B, in which the clade of the host of *Ca.* A. ciliaticola (magenta) was collapsed.**

**Figure S8 Localization and abundance of endosymbionts inside their ciliate host. Endosymbionts were visualized by FISH using oligonucleotide probe eub62A3_813 (yellow) and counterstained with DAPI (blue). DAPI staining also visualized the ciliate nuclei. Endosymbionts were either evenly dispersed throughout the ciliate host cell (upper panel) or aggregated into cell clusters (lower panel). Scale bars, 10 µm.**

**Figure S9 FISH images showing the simultaneous fluorescence labeling of plagiopylean ciliates using probe plagi_1083 (magenta, first column), *Ca.* Azoamicus using probe eub62A3_813 (yellow, second column) and DNA was stained with DAPI (blue, third column). A-D, E-H, I-L show ciliates labelled simultaneously with both probes. M-P show ciliates labelled with the FISH probe Non388 as a negative control. Negative control images were taken with the maximum laser intensity in a given channel used for the images of the simultaneous labeling. Image stacks were recorded with a confocal laser scanning microscope (Zeiss LSM 780, 63× oil objective, 1.4 numerical aperture) and are presented as maximum intensity projection. Images were recorded from samples collected from 95 m water depth in Lake Lugano’s northern basin in August 2020.**
